# Factors Associated with Increased Experience of Postoperative Pain after Laparoscopic Gastric Bypass Surgery

**DOI:** 10.1007/s11695-017-2570-4

**Published:** 2017-01-31

**Authors:** Markus Hartwig, Renée Allvin, Ragnar Bäckström, Erik Stenberg

**Affiliations:** 1 Department of Anaesthesiology, Faculty of Medicine and Health, Örebro University, Örebro, Sweden; 2 Department of Anaesthesiology and Intensive care, Örebro University Hospital, Örebro, Sweden; 3 Department of Surgery, Faculty of Medicine and Health, Örebro University, -70185 Örebro, SE Sweden

**Keywords:** Gastric bypass, Laparoscopy, Postoperative pain, Risk factor, Obesity, Sex, Pre-existing pain, Age

## Abstract

**Introduction:**

Patients with high body mass index (BMI), pre-existing pain and young age and women seem to experience more postoperative pain. Few studies have, however, addressed these risk factors amongst obese patients undergoing bariatric surgery.

The aim of the present study was to evaluate risk factors for postoperative pain following laparoscopic gastric bypass surgery.

**Methods:**

In this cohort study, we used data from the PAIN OUT register for postoperative pain during the first 24 h after surgery. Primary outcome measure was severity of pain after surgery. Multivariate analyses were conducted to evaluate BMI, young age, gender and pre-existing pain as independent risk factors for postoperative pain.

**Results:**

We included 192 patients in this study. Younger age (B −0.08, 95%CI −0.11 to −0.05/year; *p* < 0.001), female gender (B 0.92, 95%CI 0.10–1.75; *p* = 0.029) and pre-existing pain (B 1.06, 95%CI 0.03–2.09; *p* = 0.044) were all associated with an increased risk for postoperative pain. In the multivariate analyses, only young age ((adjusted OR 0.95, 95%CI 0.92–0.97/year; *p* < 0.001) and pre-existing pain (adjusted OR 2.56, 95%CI 1.09–6.00; *p* = 0.031) remained as independent risk factors for severe postoperative pain.

**Conclusion:**

Younger age and pre-existing pain are associated with severe postoperative pain during the first 24 h after laparoscopic gastric bypass surgery, whereas female gender and high BMI are not.

## Introduction

Postoperative pain is common after surgery, and there appears to be an association between postoperative pain and increased immune deficiency, reduced wound healing and the occurrence of chronic pain [1, 2]. Chronic pain has a negative impact on both patients and their relatives and also leads to a higher economic burden [3]. Reduction in the occurrence of postoperative pain results in earlier mobilisation after surgery and earlier discharge from hospital [4].

Depending on the definition of severe pain and the type of surgery, between 30 and 80% experience moderate to severe postoperative pain. Women are often reported to experience more postoperative pain than men, and following laparoscopic gastric bypass surgery, women consume more morphine than men in the immediate postoperative period [5]. Besides gender, there are several other known risk factors that may contribute to postoperative pain, the most prominent being young age [6–12], pre-existing pain [7–11, 13, 14] and obesity [6, 9]. However, not many studies consider these factors when comparing postoperative pain between men and women.

The aim of the present study was to evaluate risk factors for postoperative pain following laparoscopic gastric bypass surgery by analysing data from the PAIN OUT postoperative pain register.

## Materials and Methods

### Study Design and Samples

From February 2010 until November 2012, data on preoperative characteristics and postoperative pain during the first 24 h after surgery were recorded in a postoperative pain register [15, 16]. Patients were consecutively invited to participate in the study on postoperative day 1. All data were collected that day, when patients had been back on the surgical ward more than 6 h. The multi-item International Pain Outcome Questionnaire (IPOQ) [15, 16] was completed by each patient. The questionnaire has been described in detail elsewhere [15]. The presence of pre-existing pain was defined as a pre-existing painful condition for 3 months or more before surgery.

In the present study, we identified a cohort of patients from the register that had been operated with laparoscopic gastric bypass surgery at the University Hospital in Sweden (Örebro University Hospital). We excluded patients that had undergone another surgical procedure at the same time and those with incomplete pain ratings.

All patients were operated with a primary antecolic, antegastric laparoscopic gastric bypass procedure as described by Lönroth and Olbers [17].

### Patient Reported Outcomes

Pain intensity was rated according to VAS [18]. The primary outcome was presence of severe postoperative pain (VAS score 7–10) any time after surgery. Secondary outcomes were prevalence of low intensity maximum pain (VAS score 0–3) and “percentage of time with severe pain after surgery”. Percentage of time with severe pain as experienced by the patient was rated from 0 to 100% in steps of ten. Mild pain was defined as VAS score 0–3 [19].

### Statistical Methods

All analyses were made using SPSS version 22 (IBM Corporation, Armonk, NY, USA). Since analysis of VAS has been performed differently in previous studies, we analysed this both as a continuous variable as well as a categorical variable (presence of severe pain).

Linear regression was used for continuous variables and logistic regression for categorical variables. Based on reports from previous studies, BMI, age, pre-existing pain, and gender were entered into a multivariate linear regression model for the continuous variable and into a multivariate logistic regression model for the categorical variable. A *p* value ≤0.05 was considered to be statistically significant.

### Ethical Considerations

The study was approved by the Regional Ethics Committee and was conducted in accordance with the ethical standards of the Helsinki Declaration (6th revision).

## Results

A total of 206 patients undergoing primary laparoscopic gastric bypass surgery were identified. Seven of these were excluded due to another procedure being performed at the same time as the laparoscopic gastric bypass procedure. A further seven patients were excluded due to incomplete pain rating. The remaining 192 patients constituted our study cohort.

There were more women than men operated with laparoscopic gastric bypass surgery during the study period (Table 1). All operations were performed under general anaesthesia, and 98.4% had wound infiltration with local anaesthesia.

**Table 1:** Base-line characteristics

	*n* (%), Mean ± SD	Missing value
Gender		
Women, *n* (%)	137 (71.4%)	0
Men, *n* (%)	55 (28.6%)	0
Age, (years) mean ± SD	42.9 ± 12.3	0
BMI, (kg/m^2^) mean ± SD	40.5 ± 5.70	0
Co-morbid disease, *n* (%)	117 (60.9%)	0
Pre-existing pain ≥3 months prior to surgery, *n* (%)	30 (15.6%)	0

Operation time was on average 78.5 ± 24.6 min. High age was associated with longer operation times (age < 40 years 72.8 ± 21.6 min, age 40–60 years 80.3 ± 24.0 min, *p* = 0.033; age > 60 years 95.7 ± 31.6 min, *p* = 0.008). There were no differences in operating time with respect to gender (women 78.9 ± 25.2 min; men 77.5 ± 23.2 min; *p* = 0.717), pre-existing pain (no pre-existing pain 77.9 ± 24.2 min, pre-existing pain 81.9 ± 26.8 min; *p* = 0.414), and BMI (BMI < 45, 77.2 ± 24.9 min, BMI > 45, 84.0 ± 22.8 min; *p* = 0.132). Preoperatively, 174 patients (90.6%) received premedication according to local recommendations 60 min before surgery (1000–1330 mg paracetamol and 10 mg oxycodone), 14 patients (7.3%) received only non-opioids (paracetamol or non-steroidal anti-inflammatory drugs) and 3 patients (1.6%) received only opioids. One hundred and fifty patients (78.1%) required additional opioids on the recovery unit for postoperative pain.

### Postoperative Pain

Mean “worst pain since surgery” rating was 5.6 ± 2.7 according to VAS rating, with 86 patients (44.8%) rating their pain as severe. On average, the patients experienced severe pain for 20.6 ± 20.1% of the time after surgery. Women reported a greater percentage of time with severe pain than men did (women 23.1 ± 20.6% of time, men 14.2 ± 17.0% of time; *p* = 0.005), as did young patients (age < 40 years 27.3 ± 21.6%; age 40–60 years 16.5 ± 17.3%, *p* < 0.001; age > 60 years 11.1 ± 16.8%, *p* = 0.004; unadjusted B − 0.42%/year 95%CI −0.65 to −0.19, *p* < 0.001). No difference was reported for patients with pre-existing pain (pre-existing pain 20.1 ± 20.5%, no pre-existing pain 23.0 ± 17.6%; *p* = 0.474) or BMI (BMI < 45 kg/m^2^ 20.5 ± 20.5%, BMI > 45 kg/m^2^ 21.1 ± 18.4%, *p* = 0.857; unadjusted B −0.1%/BMI unit, 95%CI −0.52 to 0.50, *p* = 0.969). Forty-four patients (22.9%) experienced only mild pain after surgery. Men more often reported mild pain only (men 32.7%, women 19.0%; *p* = 0.040), as did the more elderly patients (age < 40 years 9.8%, age 40–60 years 30.4%, *p* = 0.001; age > 60 years 44.4%, *p* = 0.001, unadjusted OR 1.07/year, 95%CI 1.04–1.11, *p* < 0.001). There was no association between pre-existing pain (pre-existing pain 13.3%; no pre-existing pain 24.7%; *p* = 0.174) or BMI (BMI < 45 kg/m^2^, 23.2%; BMI >45 kg/m^2^, 21.6%, *p* = 0.835; OR 1.02/BMI unit 95%CI 0.96–1.08, *p* = 0.606) and mild maximum pain experienced. Women and patients with pre-existing pain and young age (unadjusted B −0.08, 95%CI −0.11 to −0.05; *p* < 0.001) rated their pain higher on the VAS (Table 2), while patients with a high BMI did not (unadjusted B −0.02, 95%CI −0.09 to 0.05; *p* = 0.563). Young age was associated with higher incidence of severe pain after surgery (OR 0.95/year 95%CI 0.93–0.97, *p* < 0.001), while BMI (OR 1.01/BMI unit, 95%CI 0.96–1.06; *p* = 0.805), whereas gender and pre-existing pain were not (Table 2).

**Table 2:** Postoperative pain

	Worst pain^a^	B^b^ (95%CI)	*p*	Severe pain^c^	OR (95%CI)	*p*
Gender						
Men	4.9 ± 2.7	Reference	0.029	19 (34.5%)	Reference	0.072
Women	5.9 ± 2.6	0.92 (0.10–1.75)		67 (48.9%)	1.81 (0.95–3.47)	
Pre-existing pain						
No	5.4 ± 2.7	Reference	0.044	68 (42.0%)	Reference	0.072
Yes	6.5 ± 2.4	1.06 (0.030–2.09)		18 (60.0%)	2.07 (0.93–4.59)	
Age						
<40	6.6 ± 2.2	Reference	Reference	48 (58.5%)	Reference	Reference
40–60	5.1 ± 2.8	−1.50 (−2.25 to −0.74)	<0.001	35 (38.0%)	0.43 (0.24–0.80)	0.007
>60	3.9 ± 2.4	−1.34 (−1.92 to −0.77)	<0.001	3 (16.7%)	0.14 (0.04–0.53)	0.004
BMI						
<45	5.5 ± 2.7	Reference	0.552	67 (43.2%)	Reference	0.373
>45	5.8 ± 2.7	0.29 (−0.67 to 1.25)		19 (51.4%)	1.39 (0.68–2.84)	

^a^According to VAS, reported as mean ± SD

^b^Regression coefficient

^c^Defined as worst pain being VAS 7–10, reported as *n* (%)

### Multivariate Analyses

When age, BMI, gender and pre-existing pain were entered into a multivariate logistic regression model, only young age (adjusted OR 0.95, 95%CI 0.92–0.97/year; *p* < 0.001) and pre-existing pain (adjusted OR 2.56, 95%CI 1.09–6.00; *p* = 0.031) remained as independent risk factors for severe postoperative pain, while BMI (adjusted OR 0.99, 95%CI 0.93–1.04/BMI unit; *p* = 0.619) and gender (adjusted OR 1.59, 95%CI 0.80–3.18; *p* = 0.184) did not.

When age, BMI, gender and pre-existing pain was entered into a multivariate linear regression model, age (adjusted B −0.09, 95%CI −0.11 to −0.06/year; *p* < 0.001), and pre-existing pain (adjusted B 1.29, 95%CI 0.32–2.24; *p* = 0.009) remained as independent risk factors, while BMI (adjusted B −0.05, 95%CI −0.05 to 0.01; *p* = 0.102), and gender (adjusted B 0.62; 95%CI −0.14 to 1.39; *p* = 0.107) did not.

## Discussion

Young age, pre-existing pain prior to surgery and female gender were all associated with greater postoperative pain experience in the univariate analyses. No association between BMI and postoperative pain was seen. Women were younger than men and more often had pre-existing pain. In the multivariate analyses, regardless of whether the VAS score was viewed categorically as severe pain or as a continuous scale, only young age and pre-existing pain remained as independent risk factors for postoperative pain following laparoscopic gastric bypass surgery.

Reduction in postoperative pain is very important for the patient and for their total experience of the surgical procedure. More important still is the association between postoperative pain and immune deficiency, wound healing and the occurrence of chronic pain [1, 2]. The reduction of postoperative pain experienced by the patient improves mobilisation [20] and ultimately reduces adverse postoperative outcome. One important measure in the effort to decrease postoperative pain is the identification of factors associated with increased risk for postoperative pain. Previous studies have reported that female gender, pre-existing pain, young age and high BMI may increase the risk for postoperative pain. In the present study, only young age and pre-existing pain were associated with increased risk for experiencing severe postoperative pain. All patients in the present study were, by definition, morbidly obese. Extremely high BMI is associated with increased risk for postoperative complications [21], and may well be associated with increased postoperative pain. There were very few patients in our study with a BMI >60 kg/m^2^. Further studies will be needed to verify the results of the present study amongst super-obese patients.

More women than men undergo laparoscopic gastric bypass surgery [22], and it is thus important to see if the increased risk for postoperative pain amongst women previously described [5] is, in fact, gender-associated or if there is another explanation. Even though further studies are needed to more fully clarify this matter, the results of the present study indicate that factors other than gender alone are of importance. Strong and independent associations were seen between young age and pre-existing pain and postoperative pain. Young age [6, 7, 9, 12, 23] as well as pre-existing pain [7, 9–14] have previously been reported to be associated with increased postoperative pain in fields of surgery other than bariatric surgery. The results of the present study show that these associations also exist in morbidly obese patients undergoing bariatric surgery and that young age and pre-existing pain are patient-specific factors that should be considered during postoperative care.

The present study has limitations. Self-reported data on pain may be influenced by other patient-related factors such as ethnicity [24] which we did not consider. If different ethnical groups have significantly different patient-specific characteristics, this could potentially have influenced our results. Our experience in Sweden, however, is that the bariatric surgery patient population is fairly stable and it is unlikely that such an unequal distribution existed. If VAS is considered as a continuous variable, even small changes may have an impact on the result. The differences in mean VAS between the groups in our study, however, were one score rating or more, which is equivalent to 10 mm or more on a 100-mm scale. Previous studies have reported that differences of 8–10 mm may be considered to be clinically relevant [18, 25, 26]. The study cohort consisted of 192 patients. It is possible that the use of a larger study group would have captured smaller effects of factors such as gender, particularly in the multivariate analyses. However, the study cohort was large enough to identify young age and pre-existing pain as independent and clinically relevant risk factors for severe postoperative pain. Smaller differences potentially detectable in a larger study cohort may not be clinically relevant. Not all patients received local anaesthesia at the time of surgery and not all patients received pre-medication according to local standards. Due to drug intolerance or previous personal experiences, however, in clinical practise, not all patients can be treated according to standard protocols. Although these deviations from standard protocols may affect experience of post-operative pain, there were no differences in use of pre-medication and local anaesthesia based on age, gender, pre-existing pain or BMI. Finally, the study was based on data collected at one point in time postoperatively and represents pain during the first 24 h after surgery only. This could lead to recall bias, but it is unlikely that this would constitute a differential bias that would affect the present results.

## Conclusion

Young age and pre-existing pain are associated with severe postoperative pain during the first 24 h after laparoscopic gastric bypass surgery. Gender appears to have less impact upon the risk for severe postoperative pain. A high BMI in these morbidly obese patients did not appear to be associated with severe postoperative pain in patients undergoing laparoscopic gastric bypass surgery.
